# Suplementação de Vitamina D Induz Remodelação Cardíaca em Ratos: Associação com a Proteína de Interação com a Tiorredoxina e a Tiorredoxina

**DOI:** 10.36660/abc.20190633

**Published:** 2021-05-06

**Authors:** Priscila P. dos Santos, Bruna P. M. Rafacho, Andrea F. Gonçalves, Vanessa C. M. Pires, Meliza G. Roscani, Paula S. Azevedo, Bertha F. Polegato, Marcos F. Minicucci, Ana Angélica H. Fernandes, Suzana E. Tanni, Leonardo A. M. Zornoff, Sergio A. R. de Paiva

**Affiliations:** 1 UNESP Faculdade de Medicina de Botucatu BotucatuSP Brasil Faculdade de Medicina de Botucatu – UNESP, Botucatu, SP - Brasil.; 2 UNESP Instituto de Biociências de Botucatu BotucatuSP Brasil Instituto de Biociências de Botucatu-UNESP, Botucatu, SP - Brasil.; 3 Centro de Pesquisa em Alimentos São PauloSP Brasil Centro de Pesquisa em Alimentos, São Paulo, SP - Brasil.

**Keywords:** Vitamina D, Remodelação Ventricular, Ratos, Tiorredoxinas, Estresse Oxidativo

## Abstract

**Fundamento:**

A vitamina D (VD) tem um importante papel na função cardíaca. No entanto, a vitamina exerce uma curva “dose-resposta” bifásica na fisiopatologia cardiovascular e pode causar efeitos deletérios, mesmo em doses não tóxicas. A VD exerce suas funções celulares ligando-se ao seu receptor. Ainda, a expressão da proteína de interação com a tiorredoxina (TXNIP) é positivamente regulada pela VD. A TXNIP modula diferentes visa de sinalização celular que podem ser importantes para a remodelação cardíaca.

**Objetivos:**

Avaliar se a suplementação com VD leva à remodelação cardíaca, e se a TXNIP e a tiorredoxina (Trx) estão associadas com esse processo.

**Métodos:**

Duzentos e cinquenta ratos Wistar machos foram alocados em três grupos: controle (C, n=21), sem suplementação com VD; VD3 (n = 22) e VD10 (n=21), suplementados com 3,000 e 10,000 UI de VD/ kg de ração, respectivamente, por dois meses. Os grupos foram comparados por análise de variância (ANOVA) com um fator e teste post hoc de Holm-Sidak (variáveis com distribuição normal), ou pelo teste de Kruskal-Wallis e análise post-hoc de Dunn. O nível de significância para todos os testes foi de 5%.

**Resultados:**

A expressão de TXNIP foi mais alta e a atividade do Trx foi mais baixa no grupo VD10. Os animais que receberam suplementação com VD apresentaram aumento de hidroperóxido lipídico e diminuição de superóxido dismutase e glutationa peroxidase. A proteína Bcl-2 foi mais baixa no grupo VD10. Observou-se uma diminuição na β-oxidação de ácidos graxos, no ciclo do ácido tricarboxílico, na cadeia transportadora de elétrons, e um aumento na via glicolítica.

**Conclusão:**

A suplementação com VD levou à remodelação cardíaca e esse processo pode ser modulado por TXNIP e Trx, e consequentemente por estresse oxidativo.

## Introdução

A Vitamina D (VD) é um composto lipossolúvel que sabidamente afeta órgãos-alvo clássicos, como ossos, intestinos e rins, e estimula o transporte de cálcio desses órgãos para o sangue.^1^ No entanto, há evidências crescentes de que a VD afeta outros órgãos, incluindo o coração, e pode exercer um importante papel na função e no desenvolvimento cardíacos.^2^^,^^3^

A prevalência da deficiência de VD aumentou nos últimos anos, tornando-se um problema de saúde pública em todo o mundo.^4^ Além disso, a deficiência de VD está associada a um risco aumentado de se desenvolver várias doenças crônicas, incluindo doenças cardiovasculares.^5^ Assim, pesquisadores têm recomendado aumento na exposição solar, fortificação de alimentos e suplementação de VD, tanto para pessoas em maior risco de hipovitaminose D como para a população geral.^6^^–^^9^ No entanto, mais estudos com diferentes doses de suplementação de VD são urgentemente necessários,^10^^–^^12^ dado o número de estudos relatando efeitos cardiovasculares deletérios da VD em doses não tóxicas.^10^^,^^11^^,^^13^^-^^15^ Estudos com ratos urêmicos e ratos infartados suplementados com VD em doses que não induzem hipercalcemia (não hipercalcêmicas), desenvolveram hipertensão,^13^ alterações na aorta,^13^ hipertrofia ventricular esquerda,^13^^,^^14^ disfunção cardíaca e alterações no metabolismo energético cardíaco.^14^ Além disso, estudo realizado com ratos normotensos mostrou que a suplementação de VD em doses não hipercalcêmicas levou ao aumento da pressão arterial e a alterações na estrutura e função vascular, mediados por geração de espécies reativas e alteração na biodisponibilidade de óxido nítrico.^15^ Esses dados indicam que a VD exerce uma curva “dose-resposta” bifásica na remodelação cardíaca.^10^

A remodelação cardíaca é causada por uma lesão no coração que pode levar a mudanças celulares, intersticiais e moleculares progressivas.^16^ As alterações celulares e moleculares incluem estresse oxidativo, apoptose, e mudança no metabolismo energético, que pode progredir para hipertrofia e disfunção ventricular.^14^

A VD exerce funções celulares ligando-se ao seu receptor e levando à regulação transcricional de genes alvos.^17^ Ainda, Chen e DeLuca^18^ identificaram um gene da proteína VDUP1 (*VD3-up-regulated protein-1*), estimulada pelo tratamento com 1,25-dihidroxivitamina D3 (1,25(OH)2D3) em células de linhagem promielocítica humana.^18^ Desde então, a VDUP1 tem sido identificada em vários tecidos, incluindo o coração.^19^

A proteína codificada pela VDUP1 é conhecida como proteína de interação com a tiorredoxina (TXNIP), e foi identificada como um regulador negativo da tiorredoxina (Trx). A TXNIP liga-se ao centro catalítico da Trx, formando um complexo estável por ligações dissulfeto, reduzindo sua atividade.^20^ Isso causa um desequilíbrio antioxidante, uma vez que o sistema Trx é um importante sistema antioxidante redutor de tiol no coração.^21^^,^^22^ De fato, estudos com células cancerosas mostraram que o tratamento com VD aumentou a produção de espécies reativas de oxigênio (EROS).^23^^–^^25^

Estudos têm mostrado que tanto a Trx como a TXNIP modulam diversas vias por meio da interação direta com moléculas de sinalização intracelular. Essas proteínas participam na regulação de vias apoptóticas e hipertróficas, e modulam o metabolismo energético tanto em cardiomiócitos como em outros tipos celulares.^21^^,^^22^^,^^26^ Assim, a suplementação com VD em doses não hipercalcêmicas poderia levar a um desequilíbrio entre TXNIP e Trx no coração, resultando em remodelação cardíaca.

Portanto, o objetivo do presente estudo foi avaliar se a suplementação com VD em doses não hipercalcêmicas leva à remodelação cardíaca, e se a TXNIP e a Trx estão associadas a esse processo.

## Materiais e métodos

### Protocolo experimental

Todos os experimentos foram realizados de acordo com as diretrizes dos Institutos Nacionais da Saúde (NIH, *National Institutes of Health*) para o uso e cuidado de animais em laboratório e aprovados pelo comitê de ética Experimentação Animal da Faculdade de Medicina de Botucatu, UNESP, São Paulo, Brasil (2008/694). Ratos machos de 250g foram alocados aleatoriamente em três grupos e alimentados com ração à base de cereal durante dois meses. Os grupos foram: 1) grupo controle (C, n=21), que não recebeu suplementação de VD [dieta baseada em cereal – Nuvilab CR1, com composição aproximada (por kg de mistura): 220g proteína; 40g gordura; 100g mineral; 80 g fibra; e 1,800 UI VD; 2) VD3 (n=22), suplementada com 3,000 UI VD/kg de ração; e 3) VD10 (n=21), suplementada com 10,000 UI de VD/kg de ração.

O tamanho amostral foi determinado com base em nossa experiência com modelos experimentais e análises. Também usamos esse tamanho amostral em um estudo prévio conduzido em nosso laboratório para avaliar a influência da suplementação com VD sobre a pressão arterial sistólica, reatividade vascular, e propriedades mecânicas.^15^ Os animais foram alocados aleatoriamente em caixas individuais. Subsequentemente, as caixas foram escolhidas aleatoriamente para compor os diferentes grupos.

Todos os animais receberam a mesma quantidade de alimentos. A suplementação de VD foi realizada adicionando-se colecalciferol (Sigma-Aldrich, St. Louis, MO, USA) diluído em óleo de milho à ração. Todos os grupos receberam 10mL de óelo de milho por quilo de ração.

O Conselho Nacional de Pesquisa (NRC, *National Research Council*) recomendou a quantidade de 1000 UI de VD por quilo de ração para os ratos.^27^ Contudo, o conselho não estabeleceu um nível máximo de ingestão. Assim, nós utilizamos uma quantidade 10 vezes superior à dose diária recomendada como nosso limite máximo de ingestão. Shepard e DeLuca^28^ demonstraram que ratos suplementados com doses acima de 1000 UI de VD/dia (~30000 UI/kg de ração) apresentaram sinais de toxicidade tais como diarreia, perda de apetite, diminuição no ganho de peso, e calcificação renal. As doses utilizadas em nosso estudo foram 4,8 e 11,8 vezes maior que as doses recomendadas para ratos e não chegaram a 1000 UI/dia. Ainda, em nosso estudo anterior,^15^ essas doses de VD não causaram sinais de toxicidade ou hipercalcemia. Assim, as doses usadas no presente estudo foram consideradas não tóxicas em termos de metabolismo do cálcio.

### Estudo ecocardiográfico

Todos os animais foram avaliados por ecocardiografia transtorácica,^29^ utilizando um equipamento disponível comercialmente (General Electric Medical Systems, Vivid S6, Tirat Carmel, Israel), equipado com um transdutor *phased-array* 5-12 MHz. Todas as medidas foram obtidas pelo mesmo observador de acordo com as recomendações da *American Society of Echocardiography* e da *European Association of Echocardiography*.^30^

Após o exame ecocardiográfico, realizou-se eutanásia dos animais com injeção intraperitoneal de tiopental sódico em dose elevada (180 mg/Kg), e os animais foram sacrificados por decapitação. Foram coletados sangue e coração dos animais.

### Avaliação de 25-hidroxivitamina D_3_ (25 (OH) D_3_) e cálcio^31^

Concentrações plasmáticas de 25 (OH) D_3_ foram determinadas por cromatografia líquida de alta resolução (HPLC) conforme descrito por Asknes,^31^ com pequena modificação. O aparelho utilizado foi o cromatógrafo Waters 2695, com detector fotodiodo Waters 2996. A quantificação de 25 (OH) D_3_ foi realizada determinando-se áreas de pico nos cromatogramas, calibrados por quantidades conhecidas dos padrões (H4014 Sigma-Aldrich, St. Louis, MO, EUA).

Concentração sérica de cálcio foi determinada pelo método arsenazo III (kit Labor Lab, SP, Brasil).

### Hidroperóxido lipídico cardíaco, enzima antioxidante e metabolismo cardíaco

Amostras do ventrículo esquerdo (200mg) foram usadas para as medidas das concentrações de proteína total e hidroperóxido lipídico (Hl) e para determinação da atividade da glutationa peroxidase (GPx), superóxido dismutase (SOD) e catalase (CAT).^14^ O metabolismo energético cardíaco foi avaliado pela atividade das enzimas 3-hidroxiacil CoA desidrogenase (HAD), fosfofrutoquinase, lactato desidrogenase (LDH), piruvato desidrogenase, citrato sintase (CS), complexo II (succinato desidrogenase) e ATP sintase. Os testes de atividade enzimática foram realizados a 20^o^C com a absorbância medida por um espectrofotômetro Pharmacia Biotech (UV/visible Ultrospec 5000 com software Swift II Applications). Todos os reagentes foram adquiridos de Sigma (Sigma-Aldrich, St. Louis, MO, EUA).

### Western blot

Western blot foi realizado para avaliar a expressão de proteínas no ventrículo esquerdo. As amostras foram separadas em gel de poliacrilamida (SDS) a 10%, e as amostras transferidas para uma membrana de nitrocelulose. A membrana foi bloqueada com leite em pó desnatado 5% e incubada com anticorpo primário (Santa Cruz Biotechnology, Inc, Europa): VDUP1 (IgG1 monoclonal de camundongo, sc271238); Trx-1 (IgG policlonal de coelho, sc20146); coativador 1-alfa do receptor gama ativado por proliferador de peroxissoma (PGC-1α – IgG polyclonal de coleho, sc13067); receptor ativado por proliferador de peroxissoma alfa (PPAR-α - rabbit polyclonal IgG, sc9000); Bcl-2 (IgG monoclonal de coelho, sc492); caspase 3 (IgG monoclonal de coelho - Cell Signaling Technology, Inc, Beverly, MA, EUA, 9664) e anticorpo secundário conjugado com peroxidase. O substrato quimioluminescente Super Signal® West Pico (Pierce Protein Research Products, Rockford, EUA) foi usado para detectar anticorpos ligados. GAPDH (IgG1 monoclonal de camundongo, Santa Cruz Biotechnology, Inc, Europa, sc 32233) foi usado para normalização.

### Teste de redução da insulina para Trx e Trx redutase (TrxR)

A atividade da Trx no coração foi determinada pelo teste de redução da insulina segundo método descrito por Yamamoto et al.,^32^ com pequena modificação. A atividade da TrxR foi determinada pelo ensaio de redução da insulina, segundo método descrito por Schutze et al.,^33^ com pequena modificação.

### Análise estatística

A normalidade dos dados foi verificada pelo teste de Kolmogorov–Smirnov. Para as variáveis com distribuição normal, os grupos foram comparados por análise de variância (ANOVA) com um fator, e análise post hoc de Holm-Sidak; os dados foram expressos em média ± desvio padrão (DP). Para as variáveis sem distribuição normal, os dados foram comparados pelo teste de Kruskal-Wallis e teste post-hoc de Dunn, e os dados foram expressos em mediana (e intervalos superiores e inferiores). As análises estatísticas foram realizadas usando o programa Sigma Stat para Windows v3.5 (SPSS Inc. Chicago, IL, USA). Para avaliar a dose resposta da VD, utilizamos o teste de tendência – *Trend test* do software GraphPad – para as variáveis com distribuição normal, e a correlação de Spearman usada para as variáveis sem distribuição normal. O nível de significância estabelecido foi de 5% em todos os testes.

## Resultados

Como observado na Tabela 1, a suplementação de VD foi efetiva, uma vez que a ingestão diária de colecalciferol foi diferente entre os três grupos, as concentrações de 25-hidroxicolecalciferol foram maiores no VD10 que em C, e o grupo VD3 apresentou um valor intermediário. Além disso, os animais que receberam ambas as doses de VD apresentaram um leve aumento nos níveis séricos de cálcio. No entanto, os grupos que receberam a suplementação de VD encontraram-se com níveis plasmáticos normais de cálcio. Essas variáveis mostraram uma resposta dose-dependente. O peso corporal e o consumo alimentar finais não foram diferentes entre os grupos e não apresentaram resposta dose-dependente.

**Tabela 1:** Peso corporal, vitamina D, consumo alimentar, cálcio sérico e 25-hidroxicolecalciferol plasmático nos grupos de animais suplementados com vitamina D e grupo controle

Variável	C	VD3	VD10	P1 Teste de comparação	P2 Teste de tendência
Peso corporal (g)	422±26,8 (21)	429±35,6 (22)	421±31,7 (21)	0,646	0,923
Consumo alimentar (g/dia)	25,7±1,54 (21)	25,9±1,91 (22)	24,9±1,98 (21)	0,166	0,154
Ingestão de VD (UI/dia)	45,5 (44,8-48,1) (21)	123 (118-128)* (22)	290 (283-310)*# (21)	<0,001	<0,001
25 (OH) D_3_ (ng/mL)	14,6 (9,40-16,4) (7)	19,0 (17,2-32,4) (7)	35,6 (33,2-37,8)* (7)	0,007	<0,001
Ca (mg/dL)	8,25±1,10 (9)	9,32±1,15* (10)	9,44±0,54* (10)	0,023	0,011

Dados expressos em média ± DP ou mediana e percentis 25 e 75. Números em parênteses indicam o número de animais em cada grupo. C: grupo controle, sem suplementação de VD; VD3 e VD10: grupos que receberam suplementação com 3000 e 10000 UI vitamina D/kg de ração, respectivamente. VD: vitamina D; 25 (OH) D3: 25-hidroxicolecalciferol plasmático; Ca: cálcio sérico. P1: valor p para teste de ANOVA com um fator ou teste de Kruskal Wallis e Holm-Sidak ou teste post hoc de Dunn; P2: p valor para teste de tendência ou correlação de Spearman. Números em negrito representam os efeitos estatisticamente significativos

* ≠ grupo C;

# ≠ grupo VD3

**Figura 1:**
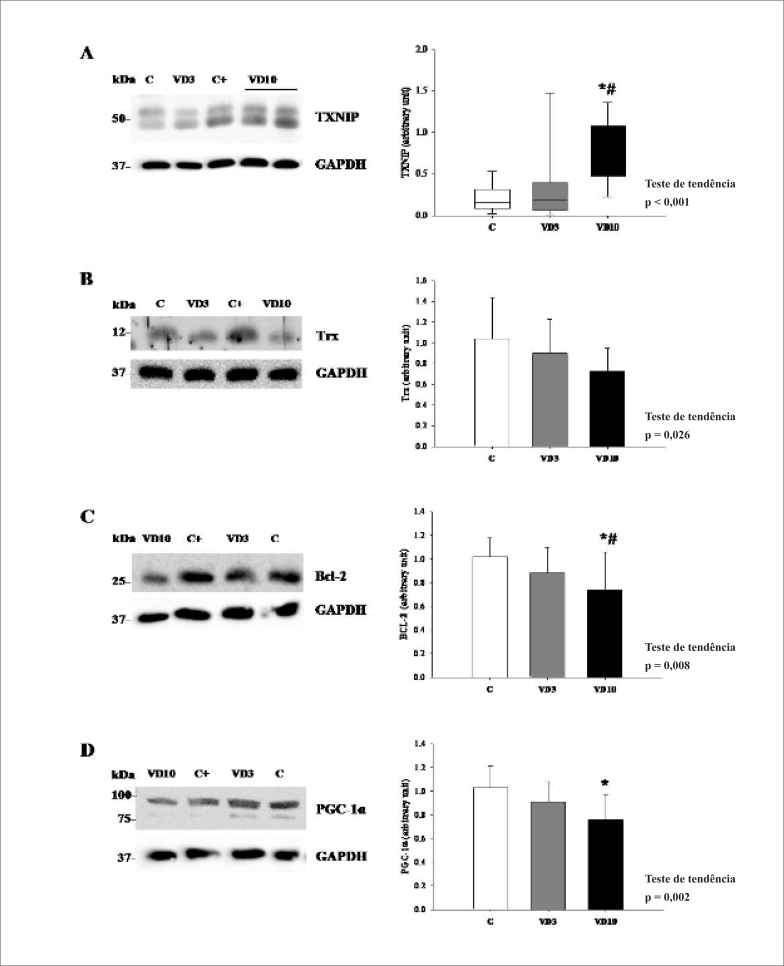
Western blot. A: Esquerda: Western blots da proteína de interação com a tiorredoxina (TXNIP). Direita: densidade de banda da razão TXNIP/GAPDH (mediana); p= 0.002. B: Esquerda: Western blots da tiorredoxina (Trx). Direita: densidade de banda da razão Trx/GAPDH (mediana); p= 0,082. C: Esquerda: Western blots da proteína Bcl-2. Direita: densidade de banda da razão Bcl-2/GAPDH (mediana); p= 0,027. D: Esquerda: Western blots do coativador 1-alfa do receptor gama ativado por proliferador de peroxissoma (PGC-1α). Direita: densidade de banda da razão PGC-1α/GAPDH (mediana); p= 0,006. Número de animais: 11-12. C: grupo controle sem suplementação de VD; VD3: grupo suplementado com 3,000 UI VD/Kg de ração; VD10: suplementado com 10 000 UI VD/Kg de ração. Análise estatística: ANOVA com um fator. * ≠ grupo C; # ≠ grupo VD3. C+ animal controle para ajuste para a corrida em gel.

Como observado nas Figuras 1A e lB e na Tabela 2, a suplementação com VD causou mudanças na TXNIP, atividade de Trx e proteína Trx, sem participação da TrxR. A expressão de TXNIP foi maior e a atividade da Trx menor no grupo VD10. Essas variáveis mostraram uma resposta dose-dependente. Ainda, observou-se uma redução na expressão de Trx de maneira dose-dependente.

**Tabela 2:** Atividade enzimática da tiorredoxina (Trx) e da tiorredoxina redutase (TrxR) nos grupos de animais suplementados com vitamina D e grupo controle

Variáveis	C	VD3	VD10	P1 Teste de comparação	P2 Teste de tendência
Atividade da Trx (DO 340 nm x minuto)	0,251±0,08 (10)	0,226±0,06 (10)	0,115±0,07*# (10)	<0,001	<0,001
Atividade da TrxR (mU/mg proteína x minuto)	0,097 (0,096-0,098) (8)	0,097 (0,096-0,097) (9)	0,096 (0,085-0,098) (10)	0,383	0,117

Dados expressos em média ± DP ou mediana e percentis 25 e 75. Números em parênteses indicam o número de animais em cada grupo. C: grupo controle, sem suplementação de VD; VD3 e VD10: grupos que receberam suplementação com 3000 e 10000 UI vitamina D/kg de ração, respectivamente. Trx: atividade enzimática da tiorredoxina; DO: densidade ótica; TrxR: tiorredoxina redutase; P1: valor p para teste de ANOVA com um fator ou teste de Kruskal Wallis e Holm-Sidak ou teste post hoc de Dunn; P2: p valor para teste de tendência ou correlação de Spearman. Números em negrito representam os efeitos estatisticamente significativos

* ≠ grupo C;

# ≠ grupo VD3.

A Tabela 3 resume dados de estresse oxidativo e apoptose. Neste estudo, os animais que receberam suplementação com VD apresentaram aumento no estresse oxidativo, demonstrado por valores mais altos de peroxidação lipídica em VD10; além disso, observamos uma atividade mais baixa das enzimas antioxidantes. As atividades de SOD e GPx foram mais baixas nos animais que receberam suplementação de VD e a razão LH/(SOD+GPx+CAT) foi mais alta no grupo VD10. Para os dados de apoptose, a expressão de proteínas antiapoptóticas Bcl-2 foi mais baixa no VD10, com resposta dose-dependente (Figura 1C). O fator apoptótico caspase-3 clivado foi mais baixo no grupo VD3 que no grupo VD10 (Tabela 3).

**Tabela 3:** Variáveis do estresse oxidativo e apoptose nos grupos de animais suplementados com vitamina D e grupo controle

Variáveis	C	VD3	VD10	P1 Teste de comparação	P2 Teste de tendência
LH (nmol/g tecido)	143,8±13,9 (8)	134,1±20,1 (8)	179,6±11,8*# (8)	<0,001	<0,001
SOD (nmol/mg proteína)	19,9 (18,6-24,5) (8)	13,0 (11,8-14,0)* (8)	13,0 (11,8-14,2)* (8)	<0,001	0,001
GPx (umol/g tecido)	40,4±6,2 (8)	31,5±4,6* (8)	29,7±3,1* (8)	<0,001	<0,001
CAT (μmol/g tecido)	120,9±15,5 (8)	124,6±11,1 (8)	110,9±15,8 (8)	0,165	0,178
LH/(SOD+GPx+CAT)	0,79±0,09 (8)	0,80±0,14 (8)	1,18±0,09*# (8)	<0,001	<0,001
Caspase-3 (unidade arbitrária)	1,01±0,49 (12)	0,84±0,48 (12)	1,54±0,56# (12)	0,023	0,060

Dados expressos em média ± DP ou mediana e percentis 25 e 75. Números em parênteses indicam o número de animais em cada grupo. C: grupo controle, sem suplementação de VD; VD3 e VD10: grupos que receberam suplementação com 3000 e 10000 UI vitamina D/kg de ração, respectivamente. SOD: superóxido dismutase; GPx: glutationa peroxidase; CAT: catalase; LH: hidroperóxido lipídico; Caspase-3: Caspase-3-clivada. P1: valor p para ANOVA com um fator e teste de Holm-Sidak ou análise post-hoc de Dunn; P2: valor p para teste de tendência ou correlação de Spearman. Números em negrito representam os efeitos significativos.

* ≠ grupo C;

# ≠ grupo VD3.

A Tabela 4 resume dados do metabolismo energético cardíaco. Em relação à β-oxidação de ácidos graxos, a expressão de PGC-1α (Figura 1D) e a atividade de HAD foram mais baixas no grupo VD10. Ambas as variáveis apresentaram uma resposta dose-dependente. Quanto à via glicolítica, a atividade das enzimas PFK e LDH foi mais elevada no grupo VD10. A enzima LDH e o complexo PDH apresentaram aumento de maneira dose-dependente. O ciclo do ácido tricarboxílico (CAT) foi avaliado pela atividade da enzima CS, e a cadeia transportadora de elétrons (CTE) avaliada pela atividade do complexo II e da atividade da ATP sintase. A atividade da CS e do complexo II foi mais baixa no grupo VD10. Ambas as enzimas apresentaram uma resposta dose-dependente. A atividade da ATP sintase variou entre os grupos, sendo maior no grupo VD3. Essas alterações indicam que os animais que receberam suplementação com VD apresentaram déficit na oxidação de ácidos graxos, no CAT e na CTE, com aumento na via glicolítica.

**Tabela 4:** Variáveis do metabolismo energético cardíaco nos grupos de animais suplementados com vitamina D e grupo controle

Variáveis	C	VD3	VD10	P1 Teste de comparação	P2 Teste de tendência
PPARα (unidade arbitrária)	1,06±0,40 (12)	0,87±0,44 (12)	0,95±0,50 (11)	0,593	0,562
HAD (nmol/mg proteína)	69,9±10,8 (8)	65,8±13,1 (8)	34,4±5,14*# (8)	<0,001	<0,001
PFK (nmol/g tecido)	131±23,6 (6)	123±34,8 (6)	170±36,4 (6)	0,048	0,053
LDH (nmol/mg proteína)	220±18,1 (8)	209±10,0 (8)	256±9,60*# (8)	<0,001	<0,001
PDH (nmol/g tecido)	317±57,9 (6)	337±42,9 (6)	382±41,6 (6)	0,088	0,034
CS (umol/g tecido)	39,7±3,22 (8)	40,4±2,75 (8)	34,5±4,02*# (8)	0,004	0,005
Complexo II (umol/mg tecido)	6,36±0,90 (6)	6,27±1,18 (6)	3,40±0,67*# (6)	<0,001	<0,001
ATP sintase (umol/mg tecido)	45,4±2,96 (6)	53,0±5,42 (6)	44,6±8,04 (6)	0,049	0,824

Dados expressos em média ±DP. Números em parênteses indicam o número de animais em cada grupo. C: grupo controle, sem suplementação de VD; VD3 e VD10: grupos que receberam suplementação com 3000 e 10000 UI vitamina D/kg de ração, respectivamente. PPARα: receptor ativado por proliferador de peroxissoma alfa; HAD: 3-hidroxiacil CoA desidrogenase; PFK: fosfofrutoquinase; LDH: lactato desidrogenase; PDH: piruvato desidrogenase; CS: citrato sintase; Complexo II: complexo II respiratório; ATP: adenosina trifosfato. P1: valor p para ANOVA com um fator e análise post-hoc de Holm-Sidak; P2: valor de p para o teste de tendência. Números em negrito representam os efeitos significativos.

* ≠ grupo C;

# ≠ grupo VD3.

Não foram observadas diferenças entre os três grupos em relação às variáveis estruturais ou na função sistólica e diastólica no ecocardiograma após dois meses de suplementação com VD. As variáveis ecocardiográficas encontram-se descritas no material suplementar (Tabela S1).

## Discussão

O presente estudo mostrou que a suplementação com VD, em doses não hipercalcêmicas durante dois meses, associou-se com maior expressão de TNIP e menor atividade da Trx. Além disso, os animais apresentaram alterações moleculares compatíveis com o processo de remodelação cardíaca, tais como estresse oxidativo, redução dos marcadores antiapoptóticos, e mudanças no metabolismo cardíaco, sem alterações na estrutura ou função cardíaca. Alterações na expressão de TXNIP e Trx podem ser um dos mecanismos envolvidos na remodelação cardíaca em animais suplementados com VD.

Um estudo prévio mostrou que a 1,25(OH)_2_D_3_ aumenta a expressão de TXNIP.^18^ A TXNIP interage com a Trx e atua como um regulador negativo de Trx, diminuindo sua expressão e sua atividade.^20^ Neste estudo, observamos que a suplementação com VD aumentou a expressão de TXNIP e reduziu a atividade de Trx. Ambas são importantes moléculas de sinalização, modulando várias funções celulares no coração, tal como o equilíbrio redox (por uma ação direta sobre EROS ou atuando sobre homeostase de proteínas e enzimas antioxidantes), apoptose e metabolismo energético.^21^^,^^22^^,^^35^ Em nosso estudo, observamos que todas essas funções celulares foram afetadas pela suplementação com VD.

Em relação ao equilíbrio redox, observamos um aumento na peroxidação lipídica e diminuição na atividade das enzimas antioxidantes SOD e GPx. Essas alterações caracterizam o estresse oxidativo.^36^ Uma diminuição nesses mecanismos antioxidantes pode induzir danos celulares graves, devido a desequilíbrios entre a produção e a remoção de radicais livres, como indicado pela razão LH/SOD+GPx+CAT no animais VD10.^37^ O sistema SOD-CAT-GPx é considerado a primeira linha de defesa contra a formação de EROS.^36^ Estudos *in vitro* (com células tumorais, adipócitos e células ósseas humanas) também mostraram um potencial papel pró-oxidante da VD. O tratamento com VD nessas células levou a mudanças no equilíbrio redox, como um aumento em EROS, e redução na SOD e glutationa.^24^^,^^25^^,^^38^

A apoptose é o processo biológico pelo qual a morte celular programada ocorre, a partir da interação de fatores pró-inflamatórios e antiapoptóticos, tal como a proteína Bcl-2.^39^ Neste estudo, mostramos uma menor expressão da Bcl-2 nos animais que receberam suplementação com VD, de maneira dose-dependente. Estudos com células tumorais também mostraram que o tratamento com VD levou ao aumento da apoptose,^24^^,^^40^ e os mecanismos envolvidos foram redução de Bcl-2^41^ e maior estresse oxidativo.^24^^,^^40^

As proteínas TXNIP e Trx participam na regulação de vias apoptóticas.^26^ Em um estudo *in vitro* realizado por Min et al.^42^ mostrou que a TXNIP diminui a expressão do gene Bcl-2. Outro estudo, com células epiteliais humanas, mostrou que o tratamento com VD aumenta a atividade de TXNIP e diminui a atividade de Trx. Além disso, os autores observaram um aumento no estresse oxidativo, diminuição na expressão de Bcl-2 e ativação da apoptose.^26^

Em nosso estudo, os animais suplementados com VD mostraram uma diminuição no fluxo de substratos oxidáveis para β-oxidação, CAT, e CTE. Por outro lado, os animais apresentaram um aumento na via glicolítica. Mudanças no metabolismo podem ser mediadas por dois importantes fatores de PGC-1α e PPAR α. O PGC-1α liga-se a PPARα e ao receptor do retinoide, formando um complexo que regula a transcrição de enzimas de β-oxidação e CTE, e inibe a oxidação do piruvato.^43^ Neste estudo, a suplementação com VD causou redução da expressão de PGC-1α. Estudos mostraram que as proteínas TXNIP e Trx regulam vias do metabolismo energético,^44^ por exemplo, modulando o PGC-1α.^45^^,^^46^ Camundongos transgênicos com expressão aumentada de Trx no coração apresentaram elevada expressão de PGC-1α e melhora na função mitocondrial.^45^^,^^46^

Nossos achados nos permitem supor que um dos mecanismos envolvidos nas alterações metabólicas e moleculares observadas nos animais suplementados com VD durante dois meses são as alterações no complmexo TXNIP/Trx.

Todas essas alterações metabólicas e moleculares precedem as alterações na estrutura e função do coração.^47^ Os animais tratados com VD por dois meses não apresentaram mudanças na estrutura ou função cardíaca. Contudo, estudos com um período mais longo de suplementação são necessários para avaliar se a VD leva a essas alterações.

Para a maioria das alterações observadas em nosso estudo, a VD mostrou uma resposta dose-dependente, e a intensidade dessas mudanças foi maior na dose mais elevada da vitamina.

### Limitações

No presente estudo, a suplementação com VD foi realizada durante dois meses, o que nos permitiu observar somente mudanças bioquímicas, celulares e moleculares. Estudos com períodos mais longos de suplementação poderiam mostrar mudanças na estrutura e função cardíaca, o que seria mais relevante clinicamente.

## Conclusão

Em nosso estudo, a suplementação com VD em doses não hipercalcêmicas levou a um processo precoce de remodelação cardíaca. O possível mecanismo das alterações cardíacas pela suplementação de VD é via modulação de TXNIP e Trx, e consequente estresse oxidativo.
