# Supplementary material for: Suplementação de Vitamina D Induz Remodelação Cardíaca em Ratos: Associação com a Proteína de Interação com a Tiorredoxina e a Tiorredoxina
Source: Arq Bras Cardiol. 2021 May 6;116(5):970–8. [Article in Portuguese] doi: 10.36660/abc.20190633 (PMC8121481; doi:10.36660/abc.20190633)
Supplement: Translation

# Translation: Vitamin D Supplementation Induces Cardiac Remodeling in Rats: Association with Thioredoxin-Interacting Protein and Thioredoxin

**Authors:** Priscila P. dos Santos, Bruna P. M. Rafacho, Andrea F. Gonçalves, Vanessa C. M. Pires, Meliza G. Roscani, Paula S. Azevedo, Bertha F. Polegato, Marcos F. Minicucci, Ana Angélica H. Fernandes, Suzana E. Tanni, Leonardo A. M. Zornoff, Sergio A. R. de Paiva

**Affiliations:** 1 BotucatuSP Brazil Faculdade de Medicina de Botucatu – UNESP, Botucatu, SP - Brazil.; 2 BotucatuSP Brazil Instituto de Biociências de Botucatu-UNESP, Botucatu, SP - Brazil.; 3 São PauloSP Brazil Centro de Pesquisa em Alimentos, São Paulo, SP - Brazil.

**Keywords:** Vitamin D, Ventricular Remodeling, Rats, Thioredoxins, Oxidative Stress

## Abstract

**Background:**

Vitamin D (VD) has been shown to play an important role in cardiac function. However, this vitamin exerts a biphasic “dose response” curve in cardiovascular pathophysiology and may cause deleterious effects, even in non-toxic doses. VD exerts its cellular functions by binding to VD receptor. Additionally, it was identified that the thioredoxin-interacting protein (TXNIP) expression is positively regulated by VD. TXNIP modulate different cell signaling pathways that may be important for cardiac remodeling.

**Objective:**

To evaluate whether VD supplementation lead to cardiac remodeling and if TXNIP and thioredoxin (Trx) proteins are associated with the process.

**Methods:**

A total of 250 Male Wistar rats were allocated into three groups: control (C, n=21), with no VD supplementation; VD3 (n = 22) and VD10 (n=21), supplemented with 3,000 and 10,000 IU of VD/ kg of chow respectively, for two months. The groups were compared by one-way analysis of variance (ANOVA) and Holm-Sidak post hoc analysis, (variables with normal distribution), or by Kruskal-Wallis test and Dunn's test post hoc analysis. The significance level for all tests was 5%.

**Results:**

TXNIP protein expression was higher and Trx activity was lower in VD10. The animals supplemented with VD showed increased lipid hydroperoxide and decreased superoxide dismutase and glutathione peroxidase. The protein Bcl-2 was lower in VD10. There was a decrease in fatty acid β-oxidation, tricarboxylic acid cycle and electron transport chain with shift to increase in glycolytic pathway.

**Conclusion:**

VD supplementation led to cardiac remodeling and this process may be modulated by TXNIP and Trx proteins and consequently oxidative stress.

## Introduction

Vitamin D (VD) is a fat-soluble compound known to affect classical target organs, like bones, intestines and kidneys, and stimulates calcium transport from these organs to the blood.^1^ However, increasing evidence has shown that VD affects other organs including the heart and may play an important role in cardiac development and function.^2^^,^^3^

The prevalence of VD deficiency has increased in recent years, becoming a public health problem worldwide.^4^ Furthermore, VD deficiency is associated with an increased risk of developing several chronic diseases including cardiovascular diseases.^5^ Therefore, researchers have recommended increased sun exposure, food fortification and VD supplementation, both for people at higher risk for hypovitaminosis D and for the general population.^6^^–^^9^ However, further research with different doses of VD supplementation is urgently needed,^10^^–^^12^ due to increasing reports of deleterious cardiovascular effects of VD in non-toxic doses.^10^^,^^11^^,^^13^^-^^15^ Uremic rats and infarcted rats, both supplemented with VD at non-hypercalcemic dosages presented hypertension,^13^ changes in the aorta,^13^ left ventricular hypertrophy,^13^^,^^14^ cardiac dysfunction, and changes in cardiac energy metabolism.^14^ Additionally, study with normotensive rats showed that VD supplementation at non-hypercalcemic doses led to increased blood pressure and changes in vascular structure and function, mediated by generation of reactive species and changes in nitric oxide bioavailability.^15^ These data indicate that VD exerts a biphasic “dose response” curve on cardiac remodeling.^10^

Cardiac remodeling is caused by an injury to the heart, which can lead to progressive cellular, interstitial, and molecular changes.^16^ The cellular and molecular alterations include oxidative stress, apoptosis, and cardiac energy metabolism change, which can progress to hypertrophy and ventricular dysfunction.^14^

VD exerts its cellular functions by binding to VD receptor and leading to transcriptional regulation of target genes.^17^ Additionally, Chen and DeLuca^18^ identified a VD3-up-regulated protein1 (VDUP1) gene that is up-regulated in the human HL-60 promyelocytic cell line by 1,25-dihydroxyvitamin D3 (1,25(OH)2D3) treatment.^18^ Since then, VDUP1 has been identified in various tissues, including the heart.^19^

The protein encoded by VDUP1 is known as thioredoxin-interacting protein (TXNIP) and was identified as a negative regulator of thioredoxin (Trx). TXNIP binds to the catalytic center of Trx forming a stable disulfide-linked complex, reducing its activity.^20^ This causes an antioxidant imbalance, since the Trx system is an important antioxidant thiol reducing system in the heart.^21^^,^^22^ Indeed, studies with cancer cell showed that VD treatment enhanced reactive oxygen species (ROS) production.^23^^–^^25^

Studies have shown that both Trx as TXNIP modulate different pathways by direct interaction with intracellular signaling molecules. These proteins participate in the regulation of apoptotic and hypertrophic pathways and modulate energy metabolism in both cardiomyocytes and other cell types^21^^,^^22^^,^^26^. Therefore, VD supplementation at non-hypercalcemic doses could lead to an imbalance of TXNIP and Trx in the heart, resulting in cardiac remodeling.

Thus, the aim of the present study was to evaluate whether VD supplementation at non-hypercalcemic doses leads to cardiac remodeling and whether TXNIP and Trx proteins are associated with this process.

## Materials and Methods

### Experimental protocol

All experiments were performed in accordance with the National Institutes of Health's Guidelines for the Care and Use of Laboratory Animals and were approved by the Ethics Committee for Animal Experimentation of the Botucatu Medical School, UNESP, São Paulo, Brazil (2008/694). Male Wistar rats of 250 g were randomly allocated into three groups and fed a cereal-based chow for two months. The groups were: 1) control group (C, n=21), with no supplementation of VD (cereal-based diet -Nuvilab CR1, with the approximate composition [kg mixture]: protein, 220g; fat, 40g; mineral, 100g; fiber, 80g and VD, 1,800 IU); 2) VD3 (n=22), supplemented with 3,000 IU VD/kg of chow; and 3) VD10 (n=21), supplemented with 10,000 IU VD/kg of chow.

The sample size was determined based on our experience with experimental models used and analyzes; it was also used on a previous study carried out in our laboratory, to assess the influence of VD supplementation on systolic arterial pressure, vascular reactivity, and mechanical properties.^15^ The animals were randomly placed in individual boxes. Subsequently the boxes are chosen at random to compose the different groups.

All animals were fed the same amount of chow. VD supplementation was performed by adding cholecalciferol (Sigma-Aldrich, St. Louis, MO, USA) diluted with corn oil, to the chow. All animal groups received 10 mL of corn oil per kg of chow.

The National Research Council recommended the amount of 1,000 IU of VD per kg of chow for the rats.^27^ However, the council has not established an upper intake level. Therefore, we used 10 times the recommended daily dose as our tolerable upper intake level. Shepard and DeLuca^28^ showed that rats supplemented with doses above 1,000 IU of VD/day (~ 30,000 IU/kg of chow) presented toxicity signs such as diarrhea, loss of appetite, decrease in weight gain, and kidney calcification. The doses used in our study were 4.8 and 11.8 times higher than the recommended dose for rats and did not reach the 1,000 IU/day. Furthermore, in our previous study,^15^ these VD doses did not cause toxicity signs or hypercalcemia. Therefore, the doses used in the present study were considered non-toxic in terms of calcium metabolism.

### Echocardiographic study

All animals were evaluated by transthoracic echocardiography,^29^ using a commercially available echocardiographic machine (General Electric Medical Systems, Vivid S6, Tirat Carmel, Israel) equipped with a 5-12 MHz phased array transducer. All measurements were obtained by the same observer according to the American Society of Echocardiography and the European Association of Echocardiography's recommendations.^30^

After the echocardiographic study, euthanasia of animals was performed with intraperitoneal injection of sodium thiopental at excessive dose (180 mg / kg), and the animals were decapitated. The blood and the hearts of animals were collected.

### Assessment of 25-hydroxyvitamin D_3_ (25 (OH) D_3_) and calcium^31^

Plasma concentrations of 25 (OH) D_3_ were measured by high performance liquid chromatography (HPLC) as described by Asknes^31^ with slight modification.^31^ The apparatus used was the Waters 2695 chromatograph with photodiode detector Waters 2996. 25 (OH/) D_3_ was quantified by determining peak areas on high-performance liquid chromatograms, calibrated against known amounts of standards (H4014 Sigma-Aldrich, St. Louis, MO, USA).

Serum concentration of calcium was measured through arsenazo III method (test kit, Labor Lab, SP, Brazil).

### Cardiac lipid hydroperoxide, antioxidant enzyme and cardiac energy metabolism

Left ventricular samples (200 mg) were used for the measurements of total protein and lipid hydroperoxide (LH) concentration and for determination of glutathione peroxidase (GPx), superoxide dismutase (SOD) and catalase (CAT) activity.^14^ Cardiac energy metabolism was assessed by 3-hydroxyacyl coenzyme-A dehydrogenase (HADH), phosphofructokinase, lactate dehydrogenase (LDH), pyruvate dehydrogenase, citrate synthase (CS), complex II (succinate dehydrogenase), and ATP synthase activities. The enzyme activity assays were performed at 25°C with the absorbance measured using a Pharmacia Biotech spectrophotometer (UV/visible Ultrospec 5000 with Swift II Applications software). All of the reagents were from Sigma (Sigma-Aldrich, St. Louis, MO, USA).

### Western blot

Western blot was performed to analyze protein expression in the left ventricle. Samples were separated on 10% SDS-polyacrylamide gel and the proteins were transferred to a nitrocellulose membrane. The membrane was blocked with 5% nonfat dry milk and incubated with primary antibody (Santa Cruz Biotechnology, Inc, Europa): VDUP1 (mouse monoclonal IgG1, sc271238); Trx-1 (rabbit polyclonal IgG, sc20146); peroxisome proliferator-activated receptor gamma coactivator 1-alpha (PGC-1α - rabbit polyclonal IgG, sc13067); peroxisome proliferator-activated receptor-α (PPAR-α - rabbit polyclonal IgG, sc9000); Bcl-2 (rabbit monoclonal IgG, sc492); caspase 3 (rabbit monoclonal IgG - Cell Signaling Technology, Inc, Beverly, MA, USA, 9664), and secondary peroxidase-conjugated antibody. Super Signal® West Pico Chemiluminescent Substrate (Pierce Protein Research Products, Rockford, USA) was used to detect bound antibodies. GAPDH (mouse monoclonal IgG1, Santa Cruz Biotechnology, Inc, Europe, sc 32233) was used for blot normalization.

### Insulin reduction assay for Trx and thioredoxin reductase (TrxR)

The activity of Trx in the heart was determined by the insulin reduction assay, according to the method described by Yamamoto et al. 2003^32^ with slight modification. The activity of TrxR in the heart was determined by the insulin reduction assay, according to the method described by Schutze et al.^33^ with slight modification.

### Statistical analysis

The normality of the data was verified by Kolmogorov–Smirnov statistical test. The groups were compared by one-way analysis of variance (ANOVA) and Holm-Sidak post hoc analysis, for variables with normal distribution, and data are expressed as mean ± standard deviation (SD). Otherwise, the groups were compared using the Kruskal-Wallis test and Dunn's post hoc analysis and the data are expressed as medians (including the lower and upper quartiles). The statistical analyses were performed using Sigma Stat for Windows v3.5 (SPSS Inc. Chicago, IL, USA). To assess the dose-response of VD, test for trend was used: Trend test of the statistical package GraphPad Prism software was used for variables with normal distribution; and the Spearman correlation used for variables without normal distribution.^34^ The significance level for all tests was 5%.

## Results

As shown in Table 1, VD supplementation was effective, since daily intake of cholecalciferol was different between the three groups, and 25-hydroxycholecalciferol concentrations were higher in VD10 than in C, and VD3 had an intermediary value. Additionally, the animals receiving both VD doses showed a slight increase in serum calcium level. However, the supplemented groups were in normocalcemic range. These variables showed a dose-dependent response. The final body weight and food consumption did not differ among groups and did not present a dose-dependent response.

**Table 1:** Body weight, vitamin D and food ingestion, serum calcium and plasma 25-hydroxycholecalciferol (OH) D3 in the groups of rats supplemented with vitamin D and control group

Variable	C	VD3	VD10	P1 Comparison test	P2 Trend test
Body weight (g)	422±26.8 (21)	429±35.6 (22)	421±31.7 (21)	0.646	0.923
Food ingestion (g/day)	25.7±1.54 (21)	25.9±1.91 (22)	24.9±1.98 (21)	0.166	0.154
VD ingestion (IU/day)	45.5 (44.8-48.1) (21)	123 (118-128)* (22)	290 (283-310)*# (21)	<0.001	<0.001
25 (OH) D_3_ (ng/mL)	14.6 (9.40-16.4) (7)	19.0 (17.2-32.4) (7)	35.6 (33.2-37.8)* (7)	0.007	<0.001
Ca (mg/dL)	8.25±1.10 (9)	9.32±1.15* (10)	9.44±0.54* (10)	0.023	0.011

Data expressed as mean±SD or median with 25 and 75 percentiles. Numbers in parentheses indicate the numbers of animals in each group. C: control group, with no supplementation of VD; VD3 and VD10: supplemented with 3,000 and 10.000 IU VD/kg of chow, respectively; VD: vitamin D; 25 (OH) D3: plasma 25-hydroxycholecalciferol; Ca: serum calcium. P1: p value of 1-way ANOVA or Kruskal Wallis and Holm-Sidak or Dunn's test post hoc analysis; P2: p value of Trend test or Spearman correlation. Bold numbers represent the significant effects that were considered.

* ≠ C group;

# ≠ VD3 group.

As shown in Figures 1A and 1B and Table 2, VD supplementation changed were TXNIP, Trx activity and Trx protein, without TrxR participation. Protein expression of TXNIP was higher and Trx activity was lower in VD10. These variables showed a dose-dependent response. Additionally, there was a decrease in the protein expression of Trx in a dose-dependent manner.

**Figure 1:** Western blot. A: Left: representative western blots showing thioredoxin-interacting protein (TXNIP). Right: median TXNIP/GAPDH ratio band density; p= 0.002. B: Left: representative western blots showing thioredoxin (Trx). Right: median Trx/GAPDH ratio band density; p= 0.082. C: Left: representative western blots showing Bcl-2. Right: median Bcl-2/GAPDH ratio band density; p= 0.027. D: Left: representative western blots showing peroxisome proliferator-activated receptor-γ coactivator 1α (PGC-1α). Right: median PGC-1α/GAPDH ratio band density; p= 0.006. Number of animals: 11-12. C: control group, with no supplementation of VD; VD3: supplemented with 3,000 IU VD/Kg of chow; VD10: supplemented with 10,000 IU VD/kg of chow. Statistical analysis 1-way ANOVA. * C group; # ≠ VD3 group. C+ is a control animal for adjustment for gel running.

**Table 2:** Thioredoxin (Trx) and thioredoxin reductase (TrxR) enzymatic activity in the groups of rats supplemented with vitamin D and control group

Variables	C	VD3	VD10	P1 Comparison test	P2 Trend test
Trx activity (OD 340 nm x minute)	0.251±0.08 (10)	0.226±0.06 (10)	0.115±0.07*# (10)	**<0.001**	**<0.001**
TrxR activity (mU/mg protein x minute)	0.097 (0.096-0.098) (8)	0.097 (0.096-0.097) (9)	0.096 (0.085-0.098) (10)	0.383	0.117

Data expressed as mean ± SD or median with 25 and 75 percentiles. Numbers in parentheses indicate the numbers of animal in each group. C: control group, with no supplementation of VD; VD3 and VD10: supplemented with 3,000 and 10.000 IU VD/kg of chow. Trx activity: thioredoxin enzymatic activity; OD: optical density; TrxR: thioredoxin reductase. P1: p value of 1-way ANOVA or Kruskal Wallis and Holm-Sidak or Dunn's test post hoc analysis; P2: p value of Trend test or Spearman correlation. Bold numbers represent statistically significant effects.

* ≠ C group;

# ≠ VD3 group.

Table 3 summarizes the oxidative stress and apoptosis data. In this study, the animals supplemented with VD presented an increase in oxidative stress, shown by the higher lipid peroxidation values in the VD10; in addition, we observed a lower activity of antioxidant enzymes. SOD and GPx activity were lower in VD-supplemented animals and the LH/(SOD+GPx+CAT) ratio was higher in VD10. These alterations in variables showed a dose-dependent response. For apoptosis data, the expression of anti-apoptotic Bcl-2 protein was lower in VD10 and showed a dose-dependent response (Figure 1-C). The apoptotic factor, caspase-3-cleaved, was lower in VD3 than in VD10 (Table 3).

**Table 3:** Variables of oxidative stress and apoptosis in the groups of rats supplemented with vitamin D and control group

Variables	C	VD3	VD10	P1 Comparison test	P2 Trend test
LH (nmol/g tissue)	143.8±13.9 (8)	134.1±20.1 (8)	179.6±11.8*# (8)	<0.001	<0.001
SOD (nmol/mg protein)	19.9 (18.6-24.5) (8)	13.0 (11.8-14.0)* (8)	13.0 (11.8-14.2)* (8)	<0.001	0.001
GPx (umol/g tissue)	40.4±6.2 (8)	31.5±4.6* (8)	29.7±3.1* (8)	<0.001	<0.001
CAT (μmol/g tissue)	120.9±15.5 (8)	124.6±11.1 (8)	110.9±15.8 (8)	0.165	0.178
LH/(SOD+GPx+CAT)	0.79±0.09 (8)	0.80±0.14 (8)	1.18±0.09*# (8)	<0.001	<0.001
Caspase-3 (arbitrary unit)	1.01±0.49 (12)	0.84±0.48 (12)	1.54±0.56# (12)	0.023	0.060

Data expressed as mean±SD or median with 25 and 75 percentiles. Numbers in parentheses indicate the numbers of animals in each group. C: control group, with no supplementation of VD; VD3 and VD10: supplemented with 3,000 and 10.000 IU VD/Kg of chow, respectively. SOD: superoxide dismutase; GPx: gluthathione peroxidase; CAT: catalase; LH: lipid hydroperoxide; Caspase-3: Caspase-3-cleaved. P1: p value of 1-way ANOVA and Holm-Sidak or Dunn's test post hoc analysis; P2: p value of Trend test or Spearman correlation. Bold numbers represent statistically significant effects.

* ≠ C group;

# ≠ VD3 group.

Table 4 summarizes data of cardiac energy metabolism. In relation to fatty acids β-oxidation, protein expression of PGC-1α (Figure 1-D) and OHADH activity were lower in VD10. Both variables showed a dose-dependent response. The protein expression of PPARα did not differ among the groups and did not present a dose-dependent response. For the glycolytic pathway, the activity of PFK and LDH enzymes showed higher values in the VD10 group. The LDH enzyme and PDH complex showed an increase in a dose-dependent manner. The tricarboxylic acid cycle (TCA) was evaluated by the activity of CS, and the electron transport chain (ETC) was evaluated by the activity of complex II and ATP synthase activity. The activity of CS and complex II was lower in VD10. Both enzymes showed a dose-dependent response. The activity of ATP synthase differed between the groups, with higher values in the VD3 group. These alterations indicate that the animals supplemented with VD presented impairment in fatty acids oxidation, TCA, and ETC, with a shift to an increase in the glycolytic pathway.

**Table 4:** Variables of cardiac energy metabolism in the groups of rats supplemented with vitamin D and control group

Variable	C	VD3	VD10	P1 Comparison test	P2 Trend test
PPARα (arbitrary unit)	1.06±0.40 (12)	0.87±0.44 (12)	0.95±0.50 (11)	0.593	0.562
OHADH (nmol/mg protein)	69.9±10.8 (8)	65.8±13.1 (8)	34.4±5.14*# (8)	<0.001	<0.001
PFK (nmol/g tissue)	131±23.6 (6)	123±34.8 (6)	170±36.4 (6)	0.048	0.053
LDH (nmol/mg protein)	220±18.1 (8)	209±10.0 (8)	256±9.60*# (8)	<0.001	<0.001
PDH (nmol/g tissue)	317±57.9 (6)	337±42.9 (6)	382±41.6 (6)	0.088	0.034
CS (umol/g tissue)	39.7±3.22 (8)	40.4±2.75 (8)	34.5±4.02*# (8)	0.004	0.005
Complex II (umol/mg tissue)	6.36±0.90 (6)	6.27±1.18 (6)	3.40±0.67*# (6)	<0.001	<0.001
ATP synthase (umol/mg tissue)	45.4±2.96 (6)	53.0±5.42 (6)	44.6±8.04 (6)	0.049	0.824

Data expressed as mean ± SD. Numbers in parentheses indicate the number of animals in each group. C: control group, with no supplementation of VD; VD3 and VD10: supplemented with 3,000 and 10,000 IU vitamin D/kg of chow, respectively. PPARα: peroxisome proliferator-activated receptor α; OHADH: 3-hydroxyacyl coenzyme-A dehydrogenase; PFK: phosphofructokinase; LDH: lactate dehydrogenase; PDH: pyruvate dehydrogenase; CS: citrate synthase; Complex II: respiratory complex II; ATP: adenosine triphosphate. P1: p value of 1-way ANOVA and Holm-Sidak post hoc analysis; P2: p value of Trend test. Bold numbers represent statistically significant effects.

* ≠ C group;

# ≠ VD3 group

No differences were observed between the three groups in relation to structural variables, or in systolic and diastolic function by echocardiogram after two months of VD supplementation. Echocardiographic variables are provided in the supplementary material (Table S1).

## Discussion

This study showed that VD supplementation, in non-hypercalcemic doses, for two months in normal rats was associated with higher expression of TXNIP and lower Trx activity. In addition, the animals presented molecular alterations compatible with the cardiac remodeling process, such as oxidative stress, decreased anti-apoptotic markers, and alterations in cardiac energy metabolism, without changes in cardiac structure and function. Changes in the expression of TXNIP and Trx may be one of the mechanisms involved in the cardiac remodeling in animals supplemented with VD.

A previous study showed that 1,25(OH)_2_D_3_ upregulates the expression of TXNIP.^18^ TXNIP interacts with Trx and acts as a negative regulator of Trx, by decreasing its expression and its activity.^20^ In this study, we observed that VD supplementation led to higher expression of TXNIP and lower activity of Trx. TXNIP and Trx are important signaling molecules, thereby modulating various cellular functions in the heart such as the redox balance (by a direct action on ROS or acting on homeostasis of proteins and antioxidant enzymes), apoptosis and energy metabolism.^21^^,^^22^^,^^35^ In our study, we observed that all these cellular functions were affected by VD supplementation.

In relation to redox balance, we observed an increase in lipid peroxidation and decrease in the activity of antioxidant enzymes: SOD and GPx. These alterations characterize oxidative stress.^36^ A decrease in these antioxidant mechanisms can induce severe cell damages due to imbalances between the production and the removal of free radicals, as indicated by the LH/SOD+GPx+CAT ratio in VD10 animals.^37^ The SOD-CAT-GPx system is considered the first line of defense against oxyradical formation.^36^ Studies *in vitro* (with tumor cells, adipocytes, and human bone cells) have also shown a potential prooxidant role of VD. Treatment with VD in these cells led to changes in redox balance, such as an increase in ROS, and SOD and glutathione reduction.^24^^,^^25^^,^^38^

Apoptosis is the biological process by which programmed cell death occurs, requiring the interaction of pro- and anti-apoptotic factors, such as the Bcl-2 protein.^39^ In this study, we showed lower expression of Bcl-2 in animals supplemented with VD, in a dose-dependent manner. Studies with tumor cells also showed that VD treatment led to increased apoptosis,^24^^,^^40^ and the mechanisms involved are decreased Bcl-2^41^ and increased oxidative stress.^24^^,^^40^

TXNIP and Trx proteins have been shown to participate in the regulation of apoptosis pathways.^26^ An *in vitro* study performed by Min et al.^42^ showed that TXNIP down-regulates Bcl-2 gene expression. Another study, with human epithelial cells, showed that treatment with VD increases TXNIP and decreases Trx activity. In addition, the authors observed increased in oxidative stress, decreased Bcl-2 expression and apoptosis activation.^26^

In our study, the animals supplemented with VD showed a decrease in the flow of oxidizable substrates for β-oxidation, TCA, and ETC. On the other hand, the animals presented an increase in the glycolytic pathway. Changes in metabolism may be mediated by two important transcription factors, PGC-1α and PPAR α. PGC-1α binds to PPARα and retinoid receptor, forming a complex that regulates the transcription of enzymes of fatty acids β-oxidation and ETC, and inhibits pyruvate oxidation.^43^ In this study, VD supplementation led to lower PGC-1α expression. Studies have shown that TXNIP and Trx proteins regulate energy metabolism pathways,^44^ for example, modulating the PGC-1α.^45^^,^^46^ Transgenic mice that overexpress Trx in the heart presented increased expression of PGC-1α and improvement of mitochondrial function.^45^^,^^46^

Our findings allow us to suppose that one of the mechanisms involved in metabolic and molecular alterations observed in animals supplemented with VD for two months are changes in the TXNIP/Trx complex.

All these metabolic and molecular changes precede the changes in the structure and function of the heart.^47^ The animals supplemented with VD for two months showed no changes in cardiac structure and function. However, studies with prolonged supplementation are necessary to assess whether VD can lead to such changes.

For most of the changes observed in our study, VD showed a dose-dependent response, and the intensity of these changes increased at the highest dose of VD.

### Limitations

VD supplementation in this study was carried out for two months, which allowed us to observe only biochemical, cellular, and molecular changes. Studies with longer supplementation periods could show changes in cardiac structure and function, which is clinically more relevant.

## Conclusion

In conclusion, in our study, VD supplementation in non-hypercalcemic doses lead to early process of cardiac remodeling. The possible mechanism of cardiac changes by VD supplementation is by TXNIP and Trx modulation and consequently oxidative stress.
